# Supplementary material for: Heat Shock Proteins in Pancreatic Cancer: Pathogenic Mechanisms and Clinical Implications
Source: Cells. 2025 Oct 18;14(20):1627. doi: 10.3390/cells14201627 (PMC12564148; doi:10.3390/cells14201627)
Supplement: Supplementary file 1 [file cells-14-01627-s001.zip › cells-3760765-supplementary.pdf]

ST1 - Supplementary Table S1. Included studies details.

**Reference**	**Phase of study**	**in vivo/ in vitro**	**Materials**	**Topic of studies**
28	Preclinical studies	in vivo	Diabetic mice (transgenic mouse model with cardiac-specific overexpression of Hsp20 and wild-type mice)	HSP20
29	Preclinical studies	in vitro	Colorectal and lung CAFs, H747, H2030, HUVEC, Ker-CT, MRC5, LIM2099, SW620, H1792, H23, CAPAN1, DANG, MIAPACA2, PSCs, RPE1, RWPE1 cell lines (cancer cell lines representing three common cancer types (lung, colorectal and pancreatic) and a representative CAF cell line from each tumor type)	HSPB6, PTGS1
30	Preclinical studies	in vitro	GEM-sensitive pancreatic cancer SW1990 cells and GEM-resistant SW1990/GEM cells	HSP27, gemcitabine
31	Preclinical studies	in vitro	human normal pancreas and pancreatic carcinoma tissue samples(pancreatic cancer cells and normal pancreatic cells)	αA-crystallin
35	Preclinical studies	in vitro, in vivo	human plasma, AsPC-1, Capan-2, BxPC-3, MIA Paca-2 cell lines, HPDE6-C7(normal pancreatic duct epithelial cell line), mice	DnaJB11, HSPA5(BiP, Grp78)
36	Preclinical studies	in vitro	pancreatic cancer cell lines - BxPC-3(WT-Bx) and MIAPaCa-2 (WT-Mia)	DNAJA1 (HSP40)
43	Preclinical studies	in vivo	Caenorhabditis elegans	HSP110, Amyloid beta
45	Preclinical studies	in vitro	Pancreatic cancer cell lines PANC-1 and BxPC-3	HSP70
47	Preclinical studies	in vitro	cancer cells isolated from patient-derived xenografts (PDX) from three PC patients who had cachexia (PCC), three early-stage lung cancer patients without cachexia (LCC) and two renal cancer patients who were not prone to cachexia (RCC)	HSP70, HSP90

48	Preclinical studies	in vivo	Mice with orthotopic tumors grown	HSP70,
			from human pancreatic cancer cell lines (AsPC-1 and BxPC-3)	HSP90
51	Preclinical studies	in vivo	caerulein-induced CP mice	HSP90 inhibitor 17AAG
55	Preclinical studies	in vitro, in vivo	the PL45 cell line, PL45 tumor-bearing mice(tumor xenografts model)	HSP90, 18F-PEGylated San A
56	Preclinical studies	in vitro, in vivo	the PL45 cell line, tumor-(tumor xenografts model) and inflammation-bearing mice	HSP90, 18F-PEGylated San A
57	Preclinical studies	in vitro, in vivo	human pancreatic cancer associated stellate cell line h-iPSC-PDAC-, mice bearing syngeneic subcutaneous(Panc02) or orthotopic (KPC-Luc) tumors	HSP90, HSP90 inhibitor XL888
58	Clinical studies	in vivo	Peripheral blood samples from AP and PDAC patients	HSP90
59	Preclinical studies	in vitro, in vivo	PBSandmouseserum,PL45 tumor-bearing mice (tumor xenografts model)	64Cu-Di-San A1, HSP90
60	Preclinical studies	in vitro, in vivo	Human pancreatic cancer cell lines AsPC1, SW1990, BxPC-3 and Panc-1, normal human pancreatic ductalepithelialcelllineHPDE6c7, tumor-bearing mice (tumor xenografts model)	HSPB1
61	Preclinical studies	in vitro	Human pancreatic cancer cell line Panc-1	HSPB2
65	Preclinical studies, clinical studies	in vitro, in vivo	PANC-1, Aspc-1, BxPc-3, and SW1990 cells, tumor-bearing mice, peripheral blood and tissue samples of patients with chemoresistant PC	HSP90α, gemcitabine
68	Preclinical studies	in vitro, in vivo	KRAS-mutated human pancreatic ductal adenocarcinoma(PDAC)and colon cancer lines, normal fibroblasts IMR90E1A, tumor-bearing mice(tumor xenografts model)	HSPA9

69	Preclinical studies,	in vitro,	Pancreatic cancer cells (PANC-1,	HSP27
	Clinical studies	in vivo	BXPC-3,ASPC-1,andCFPAC-1),<br>50 pairs of PDAC tumors and corresponding para-carcinoma tissue paraffin specimens	
70	Preclinical studies	in vitro,<br>in vivo	Pancreatic cancer cells,<br>tumor-bearing mice	HSP27
71	Preclinical studies,<br>clinical studies	in vitro	human pancreatic cancer cells (MiaPaca2,Patu-8988T,andT3M4) and human pancreatic normal epithelial cells (HPNE), pancreatic ductal adenocarcinoma (PDAC) primary tumors samples	HSP27,<br>HSP90,<br>gemcitabine
74	Preclinical studies	in vitro	theKLM1-R pancreatic cancer cell line	HSP27,<br>active hexose-correlated compound (AHCC),CUB Domain-containing Protein 1 (CDCP1)
75	Clinical studies	in vitro,<br>in vivo	paraffin-embedded tissue of patients with PDAC who underwent surgery	HSP27,<br>gemcitabine
76	Preclinical studies	in vitro	Human PDAC cell lines (PANC1, MIA-PaCa2, BxPC3, AsPC1, Capan-2,SW1990,CFPAC1and Suit2),tumor-bearing mice (xenograft)	HSP47
78	Preclinical studies	in vivo	tumor-bearing mice(xenograft)	HSP47
80	Preclinical studies	in vivo	PANC-1andMIAPaCa-2 cell lines, tumor-bearing mice (xenograft)	HSP70
81	Preclinical studies	in vitro	human pancreatic cancer cell line Panc-28, human colorectal adenocarcinoma cell line HCT116, human cervix carcinoma HeLa, human lung adenocarcinoma cell A549,human pancreatic cancer cell line MIAPaca-2	HSPA8,<br>Maslinic acid
82	Preclinical studies	in vitro,<br>in vivo	pancreatic stellate cells, non-cancerous epithelial cells,<br>pancreatic tumor specimens,<br>tumor-bearing mice	HSP70
83	Preclinical studies	in vitro	PANC-1, BxPC-3, MIA PaCa-2, Capan2,CFPA,HPNE,T-HPNE,	HSP70

			HPNE:KRASG12D cells	
84	Preclinical studies	in vitro, in vivo	PANC1,CFPAC1,MiaPaCa2, Panc2.03, GPX4−/−andPanc02 cells, tumor-bearing mice	HSPA5
85	Preclinical studies	in vitro	PANC-1 cells	HSP70, gemcitabine, melatonin
86	Clinical studies	in vivo	Patients with HCC	radiofrequency ablation (RFA)
87	Preclinical studies, clinical studies	in vitro	Human pancreatic cell linesPANC-1, Capan-2, SW1990 and BxPC-3, human pancreatic ductal epithelial cells HPDEC, pancreas tissues from radiation- or chemotherapy-naive patients who underwent radical pancreatectomy	stress-induce d phosphoprote in 1 (STIP1), HSP70, HSP90
88	Preclinical studies	in vitro	PANC-1 human pancreatic adenocarcinoma cells	Pancastatin A and B
89	Preclinical studies	in vitro	Panc-28cells, human pancreatic cancer cells	HSPA8, Maslinic acid
90	Preclinical studies	in vitro	Human PC cell lines Mia-paca2 and Capan-2	HSP90, IGF-IR
91	Preclinical studies	in vitro	Bovine serum albumin (BSA)	HSP90
92	Preclinical studies	in vitro, in vivo	Human PC cell lines HPAC and PANC-1, tumor-bearing mice	HSP90
93	Preclinical studies	in vitro, in vivo	human head and neck squamous cell carcinoma cell lines;UMSCC1, UMSCC10B, UMSCC11B, UMSCC29, UMSCC47, UMSCC74B, UMSCC92, SCCVII, MiaPaCa-2,Panc1,BxPC3 cell lines, tumor-bearing mice (xenograft)	Hsp90 inhibitors
94	Preclinical studies	in vitro, in vivo	Human umbilical vein endothelial cells (HUVECs), Human immortalized endothelial cell line EC-RF24 [24] and mouse immortalized endothelial cell line 3B-11, tumor-bearing mice	HSP90α
95	Preclinical studies	in vitro	PANC-1,AsPC-1 cell and BxPC-3 and normal pancreatic duct epithelial (hTERT-HPNE)	HSP90

96	Preclinical studies	in vitro	The HEK293EBNA(HEK) cells, the MDA-MB-231 (HTB-26), SK-BR-3(HTB-30) and MIAPaCa-2 (CRM-CRL-1420),	HSP90
98	Preclinical studies	in vitro	pancreatic cell line cultures (Panc10.05,Panc215,A6L)	HSP90 inhibitors
99	Preclinical studies	in vitro, in vivo	murine KPC4662 PDAC cells, human PDAC lines MiaPaCa-2, Panc-1,Capan-2, AsPC1,HPAF, HPAC-1, SW1990, SU.86.86, tumor-bearing mice	HSP90, trametinib
100	Preclinical studies	in vitro	Human PDAC cell line CFPAC1	HSP90
101	Preclinical studies	in vivo	tumor-bearing mice	HSP90
102	Preclinical studies	in vitro	Human PANC-1cells	HSP90
103	Preclinical studies	in vitro, in vivo	murine pancreatic cancer cell line Panc02, human pancreatic cancer cell lines BxPC-3, MIA-PaCa-2, PANC-1, and SW1990, tumor-bearing mice	HSP70, radiofrequency ablation (RFA)

ST2- Supplementary Table S2. Details of included clinical trials

Reference	NCT identifier	Phase	Design	Endpoint	PDAC-specific
104	-	1st trial-pilot study 2nd trial-phase 1	single-arm, dose-finding study	Overall survival (OS),objctive tumour response rate (ORR), time to progression (TTP)	Cohort: pancreatic adenocarcinoma
105	NCT00550004	Phase 2	multicenter, randomized, double blind, placebo controlledtrial	Primary: OS Secondary: progression free survival (PFS), CA 19-9 levels, changes in ECOG	Cohort: pancreatic adenocarcinoma
106	NCT01844817	Phase 2	randomize, double- blinded, placebo-controlled trial	Primary: OS. Secondary: PFS,ORR	Cohort: pancreatic adenocarcinoma
107	NCT00248521	Phase 1	multicenter, non- randomizd, open-label, dose-escalation study	Primary: recommended phase 2 dose (RP2D) at 28 days after treatment. Secondary: HSP90 client protein and co-chaperone changes up to 29 days after treatment, tumour response by RECIST criteria, clinical pharmacokinetic profile	Cohort: 2 pancreatic tumour patients
108	NCT00687934	Phase 1	non-randomised, open-label, single-arm, dose escalation study	Primary: Characteristics of the safety and tolerability of a once weekly administration, RP2D, pharmacokinetics, pharmacodynamics, preeliminary clinical activity (complete, partial response; stable, progressive disease ; disease control rate(DCR))	Cohort: 2pancreatic tumour patients
109	NCT01227018	Phase 2	Non-randomised, open-label, single-arm study	Primary: DCR. Secondary: best response of OS, number of patients with each worst grade toxicity, biomarker evaluation	Cohort: pancreatic adenocarcinoma
110	-	Phase 1	-	Recommended dose, toxicity profile, clinical activity(complete, partial response), pharmacokinetics, pharmacodynamics	Cohort:1pancreatic tumour patient

111	NCT00577889	Phase 2	multicenter, randomised, open-label, parallel-assignment study	Primary: six-month survival rate. Secondary: OS, time to disease progression, confirmed response rate (complete response, partial response)	Cohort: pancreatic adenocarcinoma
112	NCT01226732	Phase 1	non-randomised, open-label, single-arm study	Primary: dosedeter mination. Secondary: drug related toxicities (DRT), response rate (RR)	Cohort: 4pancreatic tumour patients
113	NCT01132625	Phase 1	multi-center, non randomised, open-label, single-arm study	Primary: maximum tolerate dose (MTD). Secondary: toxicity, efficacy assessed by RECIST, pharmacokinetics, pharmacodynamics	Cohort:2pancreatic tumour patients
114	NCT01484860	Phase 2	non randomised, open label, single-arm study	Primary: DCR. Secondary: PFS,OS, stable disease duration, safety and tolerability	Cohort: PDAC
